# An Update on Autophagy in Prion Diseases

**DOI:** 10.3389/fbioe.2020.00975

**Published:** 2020-08-27

**Authors:** Óscar López-Pérez, Juan José Badiola, Rosa Bolea, Isidro Ferrer, Franc Llorens, Inmaculada Martín-Burriel

**Affiliations:** ^1^Laboratorio de Genética Bioquímica (LAGENBIO), Instituto Agroalimentario de Aragón-IA2, Instituto de Investigación Sanitaria Aragón-IISA, Universidad de Zaragoza, Zaragoza, Spain; ^2^Centro de Encefalopatías y Enfermedades Transmisibles Emergentes (CEETE), Instituto Agroalimentario de Aragón-IA2, Instituto de Investigación Sanitaria Aragón-IISA, Universidad de Zaragoza, Zaragoza, Spain; ^3^Centro de Investigación Biomédica en Red de Enfermedades Neurodegenerativas (CIBERNED), Instituto Carlos III, L’Hospitalet de Llobregat, Barcelona, Spain; ^4^Instituto de Investigación Biomédica de Bellvitge (IDIBELL), L’Hospitalet de Llobregat, Barcelona, Spain; ^5^Departamento de Patología y Terapéutica Experimental, Universidad de Barcelona, L’Hospitalet de Llobregat, Barcelona, Spain; ^6^Department of Neurology, Clinical Dementia Center and National Reference Center for CJD Surveillance, University Medical School, Göttingen, Germany; ^7^Centro de Investigación Biomédica en Red de Enfermedades Neurodegenerativas (CIBERNED), Instituto Carlos III, Zaragoza, Spain

**Keywords:** autophagy, LC3, p62, neurodegenerative diseases, prion diseases, Creutzfeldt-Jakob disease, scrapie, therapies

## Abstract

Autophagy is a dynamic intracellular mechanism involved in protein and organelle turnover through lysosomal degradation. When properly regulated, autophagy supports normal cellular and developmental processes, whereas defects in autophagic degradation have been associated with several pathologies, including prion diseases. Prion diseases, or transmissible spongiform encephalopathies (TSE), are a group of fatal neurodegenerative disorders characterized by the accumulation of the pathological misfolded isoform (PrP^Sc^) of the physiological cellular prion protein (PrP^c^) in the central nervous system. Autophagic vacuoles have been described in experimental models of TSE and in the natural disease in humans. The precise connection of this process with prion-related neuropathology, or even whether autophagy is completely beneficial or pathogenic during neurodegeneration, is poorly understood. Thus, the biological role of autophagy in these diseases is still open to debate. During the last years, researchers have used a wide range of morphological, genetic and biochemical methods to monitor and manipulate the autophagic pathway and thus determine the specific role of this process in TSE. It has been suggested that PrP^c^ could play a crucial role in modulating the autophagic pathway in neuronal cells, and the presence of abnormal autophagic activity has been frequently observed in several models of TSE both *in vitro* and *in vivo*, as well as in human prion diseases. Altogether, these findings suggest that autophagy is implicated in prion neuropathology and points to an impairment or failure of the process, potentially contributing to the pathogenesis of the disease. Additionally, autophagy is now emerging as a host defense response in controlling prion infection that plays a protective role by facilitating the clearance of aggregation-prone proteins accumulated within neurons. Since autophagy is one of the pathways of PrP^Sc^ degradation, and drug-induced stimulation of autophagic flux (the dynamic process of autophagic degradation activity) produces anti-prion effects, new treatments based on its activation have been tested to develop therapeutic strategies for prion diseases. In this review, we summarize previous and recent findings concerning the role of autophagy in TSE.

## Introduction

Transmissible spongiform encephalopathies (TSE), or prion diseases, are a group of fatal neurodegenerative disorders that affect both humans and animals (Prusiner, 1982). Human prion diseases include kuru, the various forms of Creutzfeldt-Jakob disease (CJD), Gerstmann-Sträussler-Scheinker (GSS) syndrome, fatal familial insomnia, sporadic fatal insomnia, and the variably protease-sensitive prionopathy (Imran and Mahmood, 2011a). In animals, they include, but are not limited to, bovine spongiform encephalopathy (BSE) in cattle, classical and atypical scrapie in sheep and goats, and chronic wasting disease in cervids (Imran and Mahmood, 2011b). Ovine scrapie was the first TSE described and constitutes one of the most widely studied models of these pathologies (Pattison and Jones, 1967).

Prion diseases are characterized by a long asymptomatic incubation period and a rapidly progressing pathology that leads inevitably to death. According to the protein-only hypothesis (Prusiner, 1982), TSE are caused by the conformational conversion of native cellular prion protein (PrP^c^), which is encoded by the prion protein (*PRNP*) gene, into an infectious misfolded isoform named scrapie-associated prion protein (PrP^Sc^). Accumulation of PrP^Sc^ in the central nervous system (CNS), which is believed to be the main pathogenic event responsible for the pathological changes produced in TSE patients, induces spongiform degeneration, glial activation and neuronal loss (Wells and McGill, 1992; Wood et al., 1997). Hence, prion diseases share profound similarities with other neurodegenerative disorders associated with the accumulation of misfolded protein aggregates like Alzheimer’s disease, Parkinson’s disease and Huntington’s disease (Soto, 2003). Despite the similarities between these diseases, prions remain unique since epidemiological data support their ability to transmit under natural and experimental conditions between individuals and, to a certain extent, between species (Mays and Soto, 2016).

For years, TSE research field has focused on characterizing the underlying molecular mechanisms of the basic pathological processes involved in prion pathogenesis and neurodegeneration. Unfortunately, despite the great efforts of the researchers, these mechanisms are not completely understood. Recent evidence has emerged implicating impaired protein homeostasis as a major cause of toxicity common to prion diseases (Goold et al., 2015). Cellular homeostasis requires a proper balance between the protein degradation and synthesis to eliminate and replace proteins, respectively. In this regard, cells employ various biological mechanisms that control protein degradation, which include lysosomal routes such as the autophagic pathway (Majeski and Dice, 2004). Existing data suggest that neurons fail to recover homeostasis after exposure to PrP^Sc^, which ultimately leads to neuronal dysfunction and death by mechanisms that originate as a survival response to intracellular PrP^Sc^ accumulation (Mays and Soto, 2016). Indeed, many studies have identified autophagic dysregulation in TSE models (Boellaard et al., 1989, 1991; Liberski et al., 2004; Sikorska et al., 2004; Mok et al., 2007; Xu et al., 2012; Homma et al., 2014; Moon et al., 2016c; Llorens et al., 2017; Thellung et al., 2018; Lopez-Perez et al., 2019a, b, 2020), although the casual relationship between these observations and disease pathogenesis is currently unknown. Considering the impact of autophagy in other neurodegenerative disorders (Bursch and Ellinger, 2005; Rubinsztein, 2006), in this review we summarize, evaluate and discuss previous and recent findings concerning the role of this process, as well as the therapeutic effects of its modulation, in prion diseases.

## Autophagy

Autophagy is a fundamental pathway of cellular catabolism and recycling, in which nonessential cytoplasm and unwanted or damaged components are sequestered in vesicles and delivered to lysosomes for degradation, thus maintaining homeostatic balance (Rubinsztein, 2006). There are three different types of autophagy in mammalian cells classified according to their method of delivery to the lysosome: macroautophagy, microautophagy and chaperone-mediated autophagy (CMA) (Mizushima et al., 2008). As macroautophagy is the most prevalent and best characterized form, it is often referred to as autophagy. During macroautophagy (hereafter autophagy), portions of the cytoplasm are surrounded by an isolation membrane of unknown origin named phagophore. Elongation and fusion of the edges of the phagophore engulf the cytoplasmic material inside a double-membrane vesicle, with about 1 μm diameter, called autophagic vacuole or autophagosome. Sequestered contents can include individual, aggregated and misfolded proteins, whole organelles such as parts of the endoplasmic reticulum (ER), mitochondria and peroxisomes, and even invading pathogens. The outer membrane of the autophagosome then docks and fuses with the lysosome to form an autophagolysosome or autolysosome, where the autophagic cargo, together with the inner membrane, is degraded by lysosomal hydrolases. Eventually, the resulting macromolecules are released back into the cytosol and recycled by the cell (Yoshimori, 2004; Rubinsztein et al., 2012; Feng et al., 2014).

## Molecular Mechanisms

Autophagy is a highly conserved process from yeast to human (Reggiori and Klionsky, 2002). To date, independent genetic screens in yeast model systems, mainly in *Saccharomyces cerevisiae*, have allowed the identification of about 30 autophagy-related (*ATG*) genes, many of which have known homologs in higher eukaryotes (Bednarczyk et al., 2018). Among these *ATG* genes, one subset is required for autophagosome formation, whose corresponding encoded proteins are known as the “core autophagy machinery” (Xie and Klionsky, 2007). Autophagosome formation can be summarized in several distinct stages: induction, nucleation, expansion, fusion, and cargo degradation/recycling (Yin et al., 2016). For simplicity, we will use in this section of the review the unified yeast nomenclature when describing autophagy-related proteins.

### Induction

**FIGURE 1:**
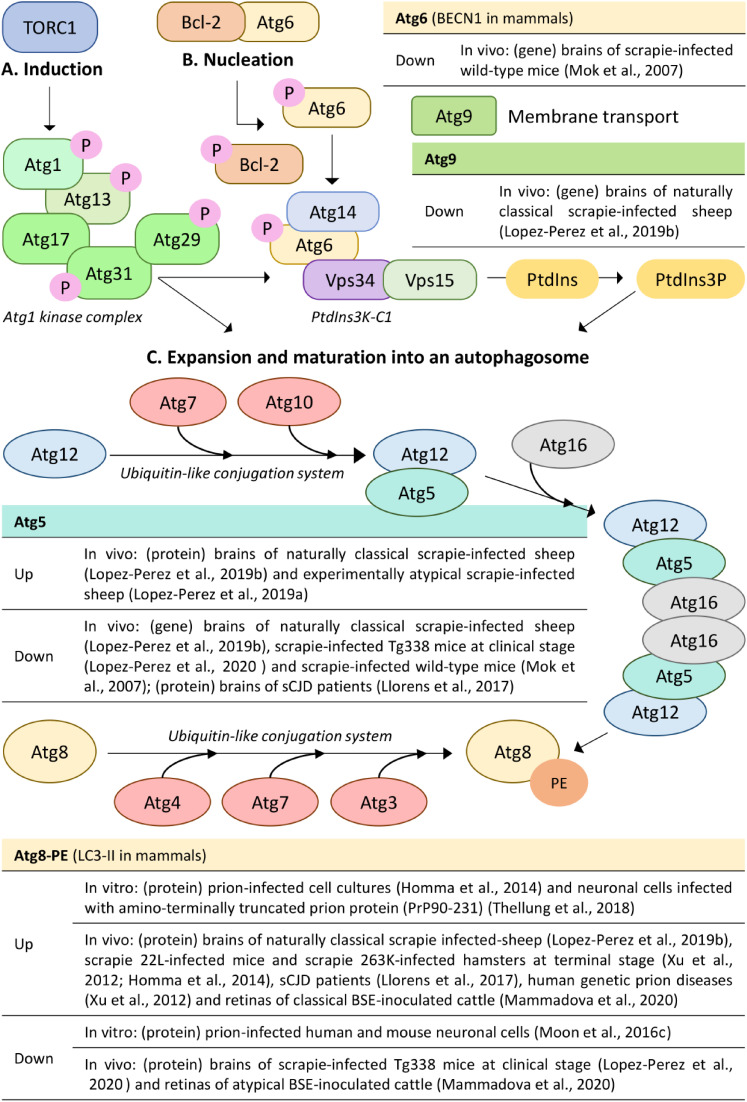
Molecular mechanisms of the core autophagy machinery and alterations (up/down) of autophagy-related genes and proteins involved in autophagosome formation in prion disease models. Figure shows the molecular mechanisms of **(A)** induction, **(B)** nucleation of the phagophore, and **(C)** expansion and maturation into an autophagosome. The unified yeast nomenclature has been used for simplicity. P, phosphate; PtdIns3K-C1, phosphatidylinositol 3-kinase complex I; PtdIns, phosphatidylinositol; PtdIns3P, phosphatidylinositol 3-phospate; PE, phosphatidylethanolamine; sCJD, sporadic Creutzfeldt-Jakob disease; BSE, bovine spongiform encephalopathy. Based on (Ariosa and Klionsky, 2016).

Initiation of the autophagic process is carried out by the Atg1 kinase complex, composed of the serine/threonine kinase Atg1, its regulatory subunit Atg13, and the Atg17-Atg31-Atg29 scaffolding subcomplex (Figure 1A; Kamada et al., 2010). The stimuli necessary to induce autophagy are detected by nutrient-sensing pathways such as the target of rapamycin complex 1 (TORC1; mTORC1 in mammals), which is considered the main negative regulator of autophagy (Laplante and Sabatini, 2012). Under normal conditions, TORC1 maintains autophagy at a basal level by phosphorylating certain proteins including Atg1 and Atg13, which impedes activation of the Atg1 kinase complex. During starvation, or treatments with chemical compounds such as rapamycin, an intracellular signaling cascade inhibits TORC1, resulting in the dephosphorylation of Atg1 and Atg13. In this situation, Atg1 auto-phosphorylates, increases its kinase activity, and recruits and activates other Atg proteins, allowing them to be localized to the phagophore assembly site (PAS) and begin nucleation (Ariosa and Klionsky, 2016).

### Nucleation of the Phagophore

In autophagy, nucleation refers to the process of mobilizing and recruitment of proteins needed for phagophore expansion to the PAS (Yin et al., 2016). The autophagy-specific class III phosphatidylinositol 3-kinase complex I (PtdIns3K-C1), which is comprised of the lipid kinase Vps34, the regulatory kinase Vps15, Vps30/Atg6 (BECN1 in mammals) and Atg14, is the nucleation machinery that is recruited to the PAS upon induction of autophagy (Figure 1B; Birgisdottir et al., 2019). The function of this set of proteins is to catalyze the conversion of phosphatidylinositol (PtdIns) to phosphatidylinositol 3-phospate (PtdIns3P). This molecule then serves as a signal that recruits other Atg proteins that recognize and preferentially bind PtdIns3P at the nucleation site (Ariosa and Klionsky, 2016). Another important negative regulator of autophagy is the Atg6-Bcl-2 interaction complex. Under normal conditions, Bcl-2 directly inhibits autophagy by binding to Atg6, while during starvation, Bcl-2 dissociates from Atg6, allowing Atg6 to associate with the PtdIns3K-C1 complex and perform its function in PtdIns3P formation (Ariosa and Klionsky, 2016).

### Expansion and Maturation Into an Autophagosome

Autophagy employs the use of two ubiquitin-like (Ubl) conjugation systems to extend and elongate the phagophore. These systems involve the Ubl proteins Atg12 and Atg8 [microtubule-associated protein 1 light chain 3 (MAP1LC3, or LC3) in mammals] (Figure 1C; Ohsumi, 2001). During the first Ubl conjugation reaction, Atg12 associates with Atg5, which depends on the E1 activating and E2 conjugating enzymes Atg7 and Atg10 (Mizushima et al., 2003b). The Atg12-Atg5 conjugate subsequently forms a multimeric complex with Atg16 (Mizushima et al., 2003a). In the second Ubl reaction, Atg8 is first cleaved off by the protease Atg4, and then recognized and processed by the E1 activating and E2 conjugating enzymes Atg7 and Atg3. The proteolyzed protein Atg8 (LC3-I in mammals) is then covalently attached to phosphatidylethanolamine (PE) to form Atg8-PE (LC3-II in mammals), a reaction that is facilitated by the E3-like Atg12-Atg5-Atg16 complex (Feng et al., 2014; Ariosa and Klionsky, 2016). Although these two conjugates are essential for autophagy and localize at the PAS, how their covalent modification reactions contribute to phagophore expansion and maturation is unknown (Ariosa and Klionsky, 2016). To further expand the phagophore, the membrane necessary is delivered from peripheral sites to the PAS by the shuttling of the transmembrane protein Atg9 (Mari et al., 2010).

### Fusion, Degradation, and Recycling

Upon completion of the autophagosome, it targets to, docks and then fuses with the degradative organelle (Yin et al., 2016). The Atg12-Atg5-Atg16 complex is mainly localized on the outer side of the autophagosome and is released into the cytosol before or after autophagosome completion (Yang and Klionsky, 2009). In contrast, Atg8-PE is present both on inner and outer autophagosome membranes (Kabeya et al., 2000). The Atg8-PE that resides on the outer side is released by a second Atg4-dependent cleavage, a deconjugation necessary to initiate the fusion stage, whereas the inner population remains inside the vesicle and is degraded in the degradative organelle (Yang and Klionsky, 2009; Nair et al., 2012). This protein is currently the most widely used marker to monitor autophagic activity because the amount of the membrane-associated LC3-II form in mammals reflects the number of autophagosomes (Mizushima and Yoshimori, 2007). After fusion, the cargo is delivered inside the degradative organelle and degraded by resident hydrolases. Metabolites generated in the proteolytic process are released back into the cytosol through various permeases located in the membrane and reutilized by the cell (Ariosa and Klionsky, 2016).

## Physiological Roles of Autophagy

Autophagy is an essential survival mechanism that primarily acts as an adaptive catabolic response to environmental adversity. This process rapidly increases its activity when cells are exposed to extreme conditions of metabolic stress, especially during nutrient deprivation, but also in the absence of growth factors, oxygen or energy (Levine and Kroemer, 2008; Kroemer et al., 2010). The basic response of autophagy is to serve as a source of energy during starvation: when there is a lack of nutrients or resources become limited, the cell is forced to break down and recycle part of its own reserves, including pre-existing material such as proteins, lipids and carbohydrates, to meet demands for metabolic substrates and stay alive until the situation improves (Levine and Kroemer, 2008).

However, constitutive turnover of cytoplasmic material by basal autophagy, even during favorable growth conditions, is also essential for proper cell physiology (Yin et al., 2016). In this regard, cells employ several quality control mechanisms aimed at degrading and recycling intracellular proteins and components to maintain normal internal homeostasis and preserve viability, which include the ubiquitin-proteasome system (UPS) and lysosomal routes such as the endocytic and autophagic pathways (Majeski and Dice, 2004). The UPS fundamentally breaks down short-lived proteins after they have executed their function in the cell (Hochstrasser, 1995), while autophagy is responsible for the degradation of long-lived proteins and whole organelles (Yoshimori, 2004). These proteolytic systems also confer a protective effect against misfolded or damaged proteins. Under normal conditions, cells efficiently control misfolded proteins by enzyme- and chaperone-mediated folding. However, when chaperone refolding fails, misfolded proteins are targeted for degradation through combined proteolytic activity of the UPS and autophagy, making interaction between these systems essential for protein quality control (Zheng et al., 2009). Existing data indicate that UPS inhibition by genetic or pharmacological manipulation induces autophagy as a protective cell response (Pandey et al., 2007) and, conversely, inhibition of autophagy increases proteasomal activity (Zhu et al., 2007), suggesting that both systems act as compensatory mechanisms in protein quality control. There are several signaling proteins that connect the UPS with the autophagic pathway, including p62 protein, or sequestosome 1 (SQSTM1), a selective autophagy receptor that recognizes and shuttles ubiquitinated proteins to the autophagosomes for degradation (Bjorkoy et al., 2005, 2006). p62 can be degraded by autophagy, since the levels of this protein decrease after treatment with autophagy inducers such as rapamycin, but increase after autophagy inhibition (Bjorkoy et al., 2005; Yue, 2007). Therefore, p62 accumulation is considered a sign of autophagic malfunction (Bjorkoy et al., 2009).

While cytosolic components and organelles are directed to lysosomes for degradation via the autophagic pathway, extracellular material and membrane proteins are directed via the endocytic pathway, thus both autophagosomes and endosomes converge in the lysosome to deliver their cargo. However, in some situations, cells relieve intracellular stress conditions by selective secretion of deleterious or damaged material, such as proteins and RNA, to the extracellular environment through exosomes (Baixauli et al., 2014). These vesicles play a crucial role in intercellular communication and, in coordination with autophagy, are essential to maintain cell homeostasis, since modulation of autophagy allows regulation of exosome biogenesis and release (Fader and Colombo, 2006). Although autophagosomes generally fuse with lysosomes, they can also fuse with multivesicular bodies containing exosomes, forming a vesicle called amphisome which subsequently fuses with lysosomes (Hessvik and Llorente, 2018). Under induction of autophagy, the multivesicular bodies target the autophagic pathway, which restrains secretion of exosomes from the cell (Fader et al., 2008).

Autophagy is also involved in other physiological processes, such as cell remodeling during development, cell differentiation, innate and adaptive immunity, genomic stability, longevity, and cell death (Levine and Yuan, 2005; Lum et al., 2005; Maiuri et al., 2007; Yorimitsu and Klionsky, 2007; Levine and Kroemer, 2008; Mizushima et al., 2008). Through all these functions, autophagy protects the organism against diverse pathologies, and when it does not work correctly, promotes the development of these. Defective autophagy has been linked to a broad range of human and animal diseases, including cardiomyopathies, infections, cancer, and protein misfolding disorders that lead to neuronal, muscle and liver degeneration (Levine and Kroemer, 2008).

## Autophagy in Neurodegenerative Diseases

Although autophagy physiologically occurs at basal level in cells, the demand for basal autophagy is tissue-specific. Those tissues whose cells do not divide after differentiation, such as neurons, myocytes or hepatocytes, are highly dependent on basal autophagy degradation, since they are predisposed to accumulation of misfolded proteins and damaged organelles that could be diluted through cell division (Komatsu et al., 2005, 2007; Hara et al., 2006; Komatsu et al., 2006; Nakai et al., 2007). In neurons, the efficient transport of proteins, organelles and autophagosomes at significant distances from the cell body through axons and dendrites (collectively known as neurites) could be altered by a defective autophagic mechanism, which would affect intercellular communication and, subsequently, contribute to neurite degeneration and neuronal cell death (Chu et al., 2009; Son et al., 2012). Additionally, neuronal autophagy has been implicated in regulation of neurite length, synapse growth and plasticity required for learning and memory (Son et al., 2012). Compared to other systems, the CNS displays low levels of autophagosomes under basal conditions and even during starvation, probably because neurons remove autophagosomes faster, or because they do not require a substantial level of autophagy due to their susceptibility to autophagic flux imbalance (Ariosa and Klionsky, 2016). The described evidence shows that basal autophagy plays a critical role in maintaining neuronal health and function, and is controlled more tightly in these cells than that of non-neuronal cells, since the intense sensitivity of the CNS to accumulation of protein aggregates and damaged organelles could easily lead to the development of neurodegenerative diseases, even in absence of expression of disease-associated mutant proteins (Frake et al., 2015; Kiriyama and Nochi, 2015).

A common feature of various neurodegenerative diseases is the intracellular accumulation of aggregates of toxic proteins that by some means escape the degradation process, which inevitable leads to dysfunction and neuronal death (Nixon, 2005; Shibata et al., 2006). The mere existence of these aggregates is a sign of malfunction of neuronal degradative mechanisms (Chu, 2011). Although most of these diseases are age-related, several *in vivo* studies have provided a direct correlation between defective autophagy and neurodegeneration. Neural-tissue deletion of *Atg* genes in mice results in accumulation of ubiquitin-positive protein aggregates in neurons, neurodegeneration, neuronal loss, progressive motor deficits and abnormal reflexes, which resemble the clinical and pathological characteristics of neurodegenerative diseases (Hara et al., 2006; Komatsu et al., 2006). Indeed, abnormal autophagic activity has frequently been observed in specific neuronal populations in Alzheimer’s disease (AD), Parkinson’s disease (PD), Huntington’s disease (HD), and prion diseases (Boellaard et al., 1989, 1991; Ravikumar et al., 2002; Qin et al., 2003; Webb et al., 2003; Liberski et al., 2004; Sikorska et al., 2004; Iwata et al., 2005; Nixon et al., 2005; Rubinsztein et al., 2005; Berger et al., 2006; Mizushima and Hara, 2006; Ventruti and Cuervo, 2007).

Autophagic vacuoles are abundant in AD brains, mainly within dystrophic neurites, but also in the cell body of affected neurons (Nixon et al., 2005). Intracellular amyloid-β (Aβ) increases autophagic activity and, in turn, autophagic vacuoles may contribute to generate the pathogenic peptide, since they are a major reservoir of intracellular Aβ and the crucial elements for its formation (Yu et al., 2005; Ling et al., 2009; Pajak et al., 2009a, b; Lipinski et al., 2010). In AD, retrograde transport of autophagosomes along microtubules and their maturation into autophagolysosomes are impaired, leading to ineffective degradation and excessive accumulation of immature autophagic vacuoles in degenerate neurites (Nixon, 2007; Boland et al., 2008). Increase or hyperphosphorylation of tau protein, which controls the stability of microtubules, may affect the activity of these tubular structures, disrupting neuronal functions of autophagic transport through neurites and synapses (Funderburk et al., 2010). In addition, the expression of ATG5, ATG12, LC3, and BECN1 decrease in AD brains as the disease progresses (Pickford et al., 2008; Ma et al., 2010). This combination of autophagy induction, defective maturation and clearance of Aβ-generating autophagosomes, and progressive reduction of key autophagy proteins, suggests an impairment of autophagic activity, which probably impedes the degradation of Aβ and creates the appropriate circumstances for its aggregation and accumulation in AD (Nixon, 2007; Boland et al., 2008; Jaeger et al., 2010).

Similarly, PD patients display characteristics of autophagy in degenerating neurons of the substantia nigra (Anglade et al., 1997). Inhibition of both CMA and macroautophagy leads to accumulation of α-synuclein (Webb et al., 2003; Cuervo et al., 2004; Vogiatzi et al., 2008), while autophagic activation through BECN1 stimulation reduces α-synuclein levels in the limbic system and improves synaptic and dendritic pathology (Spencer et al., 2009). In addition, overexpression of several familial PD-associated molecules, such as the wild-type form and the mutant forms of α-synuclein, disrupts the autophagic machinery, suggesting that autophagy may play a relevant role in the pathogenesis of PD (Cuervo et al., 2004; Xilouri et al., 2009; Winslow et al., 2010). Endosomal membranes and vacuoles with ultrastructural characteristics of autophagosomes are also increased in several experimental models of HD (Kegel et al., 2000). While wild-type huntingtin (HTT) regulates autophagy in response to ER stress, expression of its mutant form disrupts ER function and increases autophagic vacuoles (Atwal and Truant, 2008). In HD, autophagosomes form at normal rates and are adequately eliminated by lysosomes, but they fail to efficiently recognize and trap cytosolic cargo in their lumen, leading to mutant HTT accumulation in cells (Martinez-Vicente et al., 2010). Moreover, recruitment of BECN1 by accumulated mutant HTT and mutations that affect the dynein motor machinery also reduce autophagic clearance of the toxic protein (Ravikumar et al., 2005; Shibata et al., 2006).

All these observations have generated controversy regarding whether the increase of autophagosomes in degenerating neurons plays a protective homeostatic role, or instead contributes to pathogenic neuronal death (Son et al., 2012). Early studies showing accumulation of autophagosomes in brains affected with neurodegenerative diseases initially suggested that autophagy may contribute to the pathogenesis of these disorders (Levine and Kroemer, 2008). However, there is wide evidence that both Aβ peptide and the mutant forms of α-synuclein and HTT are substrates for autophagic degradation (Ravikumar et al., 2002; Webb et al., 2003; Shin et al., 2014). Induction of autophagy by pharmacological or genetic manipulation has beneficial effects and reduces the toxic protein levels in several models of neurodegenerative diseases, while its inhibition produces an opposite response (Rubinsztein et al., 2007). Therefore, the prevailing explanation is that autophagy protects against neurodegenerative diseases and the accumulation of autophagosomes produced by the expression of mutant proteins primarily represents an attempt to eliminate those proteins from the cell (Levine and Kroemer, 2008). The development of the disease implies that autophagy may reach a saturation point in which its capacity to degrade aggregated mutant proteins is exceeded, or that there are defects in the autophagic pathway produced by the same pathological factors that trigger the disease (Levine and Kroemer, 2008). In either case, impairment of autophagy likely contributes to accumulation of damaged organelles and toxic proteins, leading to the neurodegenerative conditions observed in these diseases (Son et al., 2012).

## Autophagy in Prion Diseases

Until three decades ago, data on autophagy in TSE were very limited and consisted mostly of ultrastructural studies of electron microscopy. Autophagic vacuoles in prion diseases were first described by Boellard et al. in experimental models of CJD and scrapie in mice and hamsters (Boellaard et al., 1989, 1991). Thereafter, the presence of multivesicular bodies and autophagic vacuoles was also observed in prion-infected neuronal cell cultures that displayed characteristics of neurodegeneration (Schatzl et al., 1997). Numerous large multivesicular bodies, autophagolysosomes and autophagosomes at different stages of formation have subsequently been described in neuronal cell bodies, dystrophic neurites and synapsis in brain biopsies from patients with various forms of human prion disease and in experimentally induced scrapie, CJD and GSS (Liberski et al., 2004, 2011; Sikorska et al., 2004). These findings suggest a crucial role for autophagy in TSE, which encouraged many researchers to analyze in detail this process in prion infection-related conditions. However, up to date, the role of autophagy in prion diseases is still controversial. Authors have used different approaches to quantify or monitor autophagy. Results are different *in vitro* and *in vivo* and, even in the same species, regulation can be dissimilar in different anatomical areas of the CNS. We will try to identify and discuss hereafter the origin of these discrepancies to get a global vision of autophagy studies in prion diseases.

### Is the Cellular Prion Protein Involved in Autophagy Regulation?

Although a functional role for PrP^c^ in modulating the autophagic pathway has been suggested (Shin et al., 2013), PrP^c^ deficiency does not affect basal autophagy flux of *Prnp*-knockout mice hippocampal neurons under normal culture conditions. However, under stress conditions such as serum deprivation, these cells show higher levels of LC3-II than neurons of wild-type mice (Oh et al., 2008). It was subsequently reported that autophagy regulation by PrP^c^ might be related to its known protective role against oxidative stress as the deficiency of PrP^c^ impair autophagy flux via hydrogen peroxide (H_2_O_2_)-induced oxidative stress (Oh et al., 2012), and that PrP^c^ expression increases autophagic activity through modulation of alpha7 nicotinic acetylcholine receptor (α7nAchR) to protect neurons against PrP106-126 toxicity (Jeong and Park, 2015). Thus, PrP^c^ seems to regulate autophagy only under stress conditions. Similar properties have been attributed to HTT regulating autophagy in response to ER stress (Atwal and Truant, 2008).

### Dysregulation of Autophagy in Prion Diseases

Expression studies of genes and proteins involved in the autophagic pathway have been performed in order to decipher the implication of autophagy in the neuropathology associated to TSE. Thus, the expression levels of several of these molecules are altered in several prion disease models both *in vivo* and *in vitro* (Figure 1 and Table 1). However, reported changes and their interpretation are different, and even contradictory, depending on the experimental model used.

**TABLE 1:** Alterations (up/down) of autophagy-related genes and proteins not involved in autophagosome formation in prion disease models.

Molecule	Regulation	Prion disease model	References
p62	Up	*In vitro*: (protein) prion-infected cell cultures and neuronal cells infected with amino-terminally truncated prion protein (PrP90-231)	Homma et al., 2014; Thellung et al., 2018
		*In vivo*: (protein) brains of naturally classical scrapie-infected sheep, experimentally atypical scrapie-infected sheep, scrapie-infected Tg338 mice at clinical stage, scrapie 22L-infected mice and scrapie 263K-infected hamsters	Homma et al., 2014; Lopez-Perez et al., 2019a, b, 2020
	Down	*In vitro*: (protein) prion-infected human and mouse neuronal cells	Moon et al., 2016c
		*In vivo*: (protein) brains of scrapie 263K-infected hamsters at terminal stage and human genetic prion diseases	Xu et al., 2012
FBXW7	Up	*In vitro*: (protein) prion-infected SMB-S15 cells at early stage	Xu et al., 2016
		*In vivo*: (protein) brains of scrapie 263K-infected hamsters	Xu et al., 2016
	Down	*In vivo*: (gene) brains of scrapie-infected Tg338 mice at clinical stage	Lopez-Perez et al., 2020
GAS5	Down	*In vivo*: (gene) brains of scrapie-infected Tg338 mice at clinical stage	Lopez-Perez et al., 2020
SCRG1	Up	*In vivo*: (gene) brains of scrapie- and BSE-infected mice and sCJD patients; (protein) brains of scrapie-infected mice at terminal stage	Dandoy-Dron et al., 1998, 2000; Dron et al., 1998, 2005, 2006
HSPA8, HSPB8	Up	*In vivo*: (gene) brains of sCJD-infected Tg340 mice at clinical stage, sCJD patients and naturally classical scrapie-infected sheep	Serrano et al., 2011; Llorens et al., 2017
PINK1, Parkin	Up	*In vitro*: (gene and protein) prion-infected SMB-S15 cells	Gao et al., 2020
		*In vivo*: (protein) brains of scrapie 139A- and ME7-infected mice at terminal stage	

*BSE, bovine spongiform encephalopathy; sCJD, sporadic Creutzfeldt-Jakob disease.*

Downregulation of mRNA transcripts of positive gene regulators of autophagy such as *BECN1*, *ATG5*, *ATG9*, *FBXW7* (F-box and WD repeat domain containing 7) and *GAS5* (growth arrest-specific 5) has been observed in the CNS of scrapie-infected wild-type or transgenic mice and sheep naturally affected with classical scrapie (Mok et al., 2007; Lopez-Perez et al., 2019b, 2020). These changes were associated with an impairment of autophagy at late stages of the disease, as only animals displaying clinical signs showed altered expression profiles. On the contrary, the encoded protein of *FBXW7* is upregulated at the early stage of infection in the scrapie murine brain cell line SMB-S15 and in brains of scrapie-agent 263K-infected hamsters, leading to degradation of mTORC1 and increase of autophagic flux (Xu et al., 2016). In these cells, knockdown of *ATG5* and *FBXW7* inhibited autophagic flux and increased PrP^Sc^ accumulation. Both *in vivo* and *in vitro* models suggest that downregulation of autophagy regulator genes could inhibit the process and favor/stimulate prion accumulation.

However, studies analyzing other molecular pathways related to autophagy report an increase of autophagy activity at late stages of the disease. For example, mitochondrial dysfunction is a common and prominent feature of prion diseases (Zhu et al., 2018). Mitophagy, which is the specific autophagic elimination of defective mitochondria, is activated in prion-infected SMB-S15 cells and in mice infected with scrapie strains 139A and ME7 at the terminal stage of the disease, represented by the transcriptional and protein increase of the two essential elements regulating mitophagy activity: PINK1 (PTEN-induced kinase 1) and Parkin (Gao et al., 2020). Transcripts of *SCRG1* (scrapie-responsive gene 1), whose encoded protein has been linked to the presence of autophagic vacuoles in neurons of scrapie-infected mice at terminal stage, are also enhanced in sporadic CJD (sCJD) and murine models of scrapie and BSE (Dandoy-Dron et al., 1998, 2000; Dron et al., 1998, 2005, 2006). Similarly, autophagy activator genes *HSPA8* [heat shock protein family A (Hsp70) member 8] and *HSPB8* [heat shock protein family B (small) member 8] are upregulated in the CNS of sCJD-infected Tg340-*PRNP*129MM mice at clinical stage, sCJD patients (Llorens et al., 2017), and naturally scrapie-infected sheep (Serrano et al., 2011). Upregulation of these genes involved in autophagy regulation may reflect an attempt of the cell to induce autophagy, although over time, this process may become impaired, resulting in the accumulation of autophagic vacuoles as observed in other neurodegenerative diseases (Polajnar and Zerovnik, 2014).

Nevertheless, drawing conclusions from expression changes of genes involved in the complex autophagy machinery is complicated. Universal autophagy markers could help in this analysis. The study of p62 protein, in combination with other assays such as the monitoring of LC3-II conversion, is widely used to evaluate autophagic activity (Klionsky et al., 2016; Zhang et al., 2016). Downregulation of the amount of both LC3-II and p62 in human and mouse neuronal cells infected with human prions was related to an activation of the autophagic flux in response to prion infection (Moon et al., 2016c). Interestingly, overexpression of LC3-II and p62 in prion-infected cell cultures and in brains of 263K-infected hamsters and 22L-infected mice was also associated with an activation of autophagy to promote PrP^Sc^ degradation (Homma et al., 2014). Similarly, the increment of these proteins was also observed in neuronal cells treated with amino-terminally truncated prion protein (PrP90-231), which suggested that PrP stimulates autophagic flux but leads progressively to the accumulation of autophagolysosomes with impaired resolution ability (Thellung et al., 2018). The opposite results about LC3/p62 regulation could be the consequence of analyzing different points of autophagy kinetics, or maybe TSE models are reacting differently to different prion strains. In addition, most of these studies were performed using neuronal cells and very few have focused on analyzing autophagic changes in glial cells. In fact, astrocyte reactivity resulting from activation of the unfolded protein response has been shown to be crucial in mediating prion neuropathology (Smith et al., 2020). We observed p62 immunolabeling in both neuronal and glial cells in scrapie-infected sheep and mice (Lopez-Perez et al., 2019b, 2020), but more studies including these cell populations are needed in order to deciphering the biological relevance of these findings.

Murine models allow the investigation of preclinical stages of the disease. Using mice overexpressing the highly sensitive VRQ/VRQ variant of the ovine *PRNP* gene, we described a downregulation of LC3 and upregulation of p62 in highly prion-affected brain areas of scrapie-infected Tg338 mice at clinical stage (Lopez-Perez et al., 2020). The absence of changes in scrapie-infected mice at preclinical stage indicates that impairment of autophagy takes place at the last stage of prion infection and is not the result of depletion or exhaustion of the autophagic machinery. In accordance to this hypothesis, the expression of autophagy related genes was neither altered at early stages of the disease. However, these results differ from those observed in other murine models. Conversion of LC3-I into LC3-II and downregulation of p62 have been reported in the terminal stage of scrapie 263K-infected hamsters (Xu et al., 2012). Changes reported in hamsters were similar to those observed in patients with genetic prion diseases (Xu et al., 2012). As PrP^c^ seems to have a role in autophagy regulation under stress conditions, overexpression of PrP^c^ in transgenic mice could alter the response.

Models can react differently to prion infection, but could autophagy also be regulated differently depending on the prion strain? Analyzing the CNS of sheep naturally infected with classical scrapie at clinical stage, we observed changes compatible with induction of autophagy in brain regions with low levels of prion-related lesions, such as basal ganglia and cerebellar Purkinje cells, which displayed an upregulation of both p62 and LC3 proteins (Lopez-Perez et al., 2019b). In these animals, p62 was overexpressed in most of the CNS areas. However, in brains from sheep experimentally infected with atypical scrapie at clinical stage, LC3 levels did not change and p62 was only increased in the areas most affected by prion toxicity (Lopez-Perez et al., 2019a). In addition, p62 positively correlated both with histopathological lesions and PrP^Sc^ deposits in the latter model. Considering the accumulation of p62 as a marker for autophagy malfunction, these results indicate that impairment of autophagy is limited to brain areas displaying high levels of prion-related lesions in atypical scrapie, unlike the classical form of the disease where this impairment seems to occur along the brain. The differences observed between the two forms of scrapie could reflect differences in the toxicity of both prions, as atypical scrapie displays longer incubation periods than the classical form. In line with these findings, a recent study reported that strain-dependent incubation period is negatively correlated with PrP^Sc^ deposition and neuroinflammation, but positively with autophagy (Mammadova et al., 2020). Retinas from cattle inoculated with classical BSE displayed an upregulation of autophagy indicated by the increase of LC3-II, whereas those inoculated with atypical BSE displayed a downregulation, which suggests that autophagic dysfunction contributes to increased PrP^Sc^ accumulation in atypical BSE, leading to greater neuroinflammation, shorter incubation periods and, therefore, an accelerated disease progression when compared to classical BSE.

Autophagic impairment has also been suggested in sCJD cases, where LC3-II and DJ-1 (protein deglycase DJ-1) proteins are overexpressed, while ATG5 is downregulated and LAMP2 (lysosome-associated membrane protein 2) is unaltered (Llorens et al., 2017). These alterations, along with the accumulation of autophagic vacuoles, abnormal lysosomes and autophagolysosomes observed in neurons of sCJD patients, suggest that, although autophagy mechanisms may be activated in sCJD, this process is not fully functional. Therefore, autophagic impairment does occur in prion diseases. Despite the discrepancies observed between the different TSE models, most of the reported results suggest a dysfunction of autophagy at the last stage of the disease, although it is still unknown whether such dysregulation is cause or consequence of prion toxicity.

### Autophagy in Prion Neuropathology

Despite the numerous studies reporting an abnormal autophagic activity, the biological role of this process in prion diseases, as in most neurodegenerative diseases, is currently unclear. Initially, it was suggested its participation in prion neuropathology by contributing to formation of spongiform changes (Liberski et al., 2002, 2004, 2008, 2011). Although the histogenesis of typical TSE vacuoles has yet to be explained, it has been proposed that vacuolization is by some means related to tissue destruction by autophagy, thus these vacuoles may originate abruptly from autophagic vacuoles with no detectable transition stages (Liberski et al., 2008, 2011). Excessive PrP^Sc^ accumulation could lead to overload of the catabolic machinery, phagocytosis of neuronal cytoplasm and, finally, massive elimination of damaged neurons by autophagy (Jeffrey et al., 1992). The pathogenesis of dystrophic neurites, another constant neurodegenerative alteration in TSE, is also unclear. It has been suggested that autophagic vacuoles originate in the neuronal cell body, flow down with axonal transport along the neurites, and then, in the presence of an impairment of that transport, are accumulated forming dystrophic neurites (Liberski et al., 2011; Liberski, 2019), a reasoning which resembles that described in AD (Nixon, 2007; Boland et al., 2008).

Autophagy could also be a positive factor for prion propagation. A moderate basal level of autophagy may promote prion spreading during certain stages of infection by generating smaller PrP^Sc^ seeds (Heiseke et al., 2010), which are more efficient templates for the conversion of PrP^c^ into PrP^Sc^ than larger aggregates (Silveira et al., 2005). In addition, proteasome impairment in prion-infected neuronal cells results in formation of large cytosolic perinuclear aggresomes containing PrP^Sc^ (Kristiansen et al., 2005). Similar structures were found in brains of prion-infected mice. PrP^Sc^ aggresomes may generate prion seeds (Cohen and Taraboulos, 2003), trigger autophagy (Kopito, 2000; Garcia-Mata et al., 2002) and, in turn, be sequestered by autophagosomes that fuse with lysosomes containing PrP^Sc^, where the nucleation process of forming more PrP^Sc^ can be initiated or perpetuated (Liberski et al., 2008).

In contrast to this disease-promoting role, it is also likely that the increment of autophagic vacuoles observed in prion disease models is due to the activation of the autophagic machinery as a defense mechanism that reflects the effort of neurons to survive in the harmful environment produced by PrP^Sc^ accumulation, leading even to degradation of prions (Heiseke et al., 2010). PrP^c^ is a plasma membrane-anchored glycoprotein that cycles between the cell surface and intracellularly via endosomal vesicles (Vey et al., 1996; Peters et al., 2003). After internalization, PrP^c^ traffics to early endosomes where it is directed to recycling endosomes to be returned to the plasma membrane or to multivesicular bodies to be degraded by lysosomes (Campana et al., 2005). PrP^Sc^ also appears to traffic along the same endo-lysosomal pathway (Yim et al., 2015). There are two main PrP^Sc^ populations within the infected cell: a population on the cell surface consisting of newly formed PrP^Sc^, which is highly labile, metabolizes rapidly and is a substrate for non-autophagic lysosomal degradation (Caughey and Raymond, 1991; Goold et al., 2013), and an internalized population comprising most of total cellular PrP^Sc^, which is more aggregated, is relatively stable and is subject to autophagic and proteasomal degradation (Boellaard et al., 1991; Caughey and Raymond, 1991; Heiseke et al., 2010; Goold et al., 2013; Yao et al., 2013). While implicated in the clearance of disease-associated proteins, including PrP^Sc^, degradation by the UPS may be restricted to soluble misfolded proteins or smaller oligomeric forms (Li et al., 2010; Goold et al., 2013, 2015; Bhat et al., 2014). For larger, more insoluble aggregates, the catalytic chamber of the proteasome may remain inaccessible, preventing their effective degradation (Qin et al., 2003; Scotter et al., 2014). In fact, PrP^Sc^ does not seem to be ubiquitinated to a significant degree and its status as a proteasome substrate remains controversial (Goold et al., 2015). Several studies have shown that prion infection inhibits UPS activity, likely by direct interaction between PrP^Sc^ and the 20 S proteasome core particle (Kristiansen et al., 2007; Deriziotis et al., 2011; McKinnon et al., 2016). In the context of UPS impairment, an upregulation of autophagy has been described, which may facilitate the clearance of larger aggregates (Korolchuk et al., 2010; Goold et al., 2015). Indeed, PrP^Sc^ colocalizes with lysosomal markers *in vivo* (Dearmond and Bajsarowicz, 2010) and, despite PrP^Sc^ deposits do not colocalize with autophagosomes in brains of scrapie infected hamsters, they do colocalize in prion-infected SMB-S15 cells after treatment with bafilomycin A_1_ (Xu et al., 2012). In addition, although the more usual places of PrP^Sc^ accumulation are the plasma membrane and the endosomal vesicles (Mays and Soto, 2016), and the majority of intracellular PrP^Sc^ is found within the endo-lysosomal system (Goold et al., 2015), some PrP misfolded forms harboring similarities with infectious PrP^Sc^ also appear to be present at the ER and cytosol (Mays and Soto, 2016). Since the ER lumen lacks degradation machinery, misfolded proteins are retro-translocated to the cytosol for degradation and, eventually, irreversibly aggregated ER proteins are targeted for lysosomal degradation via autophagic pathways (Goold et al., 2015). Hence, it is assumed that autophagy may be used by the cell to control or counteract prion infection providing a neuroprotective effect, while lysosomal dysfunction in combination with reduced autophagy may contribute to the development of the disease (Mok et al., 2007; Yao et al., 2013).

### Autophagy in the Treatment of Prion Diseases

At present there are no effective therapeutic or prophylactic treatments for prion diseases, nor any useful drugs are available. Existing data indicate that neurons do not recover cell homeostasis after exposure to PrP^Sc^, which eventually leads to cell damage and neuronal cell death (Mays and Soto, 2016). Considering the complex pathogenesis of TSE, with many factors contributing to toxicity (Aguzzi and Falsig, 2012), treatments aimed at the basic toxic cause, that is, PrP^Sc^ accumulation, should be effective in improving all aspects of toxicity (Goold et al., 2015). There is wide experimental evidence that induction of autophagy by chemical compounds has beneficial effects and results in decreased PrP^Sc^ both *in vitro* and *in vivo*, indicating that the stimulation of this degradative pathway is sufficient to overcome the apparent stability of internal PrP^Sc^ levels, whereas inhibition of the process by pharmacological blocking or gene silencing leads to prion accumulation (Heiseke et al., 2010; Goold et al., 2015; Abdelaziz et al., 2019).

Imatinib increases lysosomal clearance of PrP^Sc^ in cultured prion-infected cells by activating the autophagic machinery (Ertmer et al., 2004, 2007). Both lithium and trehalose also improve PrP^Sc^ clearance in prion-infected neuroblastoma and neuronal cells, respectively, by inducing autophagy (Aguib et al., 2009; Heiseke et al., 2009). Treatment with lithium not only decreases PrP^Sc^ levels, but also those of PrP^c^, which indirectly contributes to PrP^Sc^ reduction by limiting the amount of substrate available for prion conversion (Heiseke et al., 2010). Both rapamycin and metformin, which are known to inhibit mTORC1 signaling, also decrease the PrP^Sc^ load in prion-infected neuroblastoma and neuronal cells, respectively, by enhancing autophagy, suggesting that both mTORC1-dependent and independent autophagy induction pathways are involved in prion degradation (Heiseke et al., 2009; Ishibashi et al., 2015; Abdelaziz et al., 2020). In addition, both PrP^c^ and PrP^Sc^ are actively released into the extracellular environment in association with exosomes, contributing to the spread of prion infectivity between cells (Fevrier et al., 2004). Autophagy stimulation with rapamycin strongly inhibits exosomal prion release and hence the intercellular prion dissemination, while its inhibition promotes the release of exosomes and exosome-associated prions (Abdulrahman et al., 2018). Natural compounds such as hinokitiol and Ginsenoside-RG3, and other chemical compounds such as AR-12, spermine, resveratrol, FK506 and astemizole, also have anti-prion effects *in vitro* by induction of autophagy (Jeong et al., 2012; Karapetyan et al., 2013; Nakagaki et al., 2013; Moon et al., 2016a, b; Wang et al., 2016; Abdulrahman et al., 2017; Phadwal et al., 2018).

Some of these treatments are effective also *in vivo* both in terms of PrP^Sc^ reduction and beneficial clinical effects. Intraperitoneal or oral application of trehalose, rapamycin, imatinib, astemizole, AR-12 or FK506 to prion-infected mice during the early stage of infection produces anti-prion effects, such as extension of incubation period and survival time, and delay of PrP^Sc^ neuroinvasion and the onset of clinical signs, by induction of autophagosome formation and autophagy (Yun et al., 2007; Aguib et al., 2009; Heiseke et al., 2009; Cortes et al., 2012; Karapetyan et al., 2013; Nakagaki et al., 2013; Abdelaziz et al., 2019). However, the administration of some of these drugs when neuroinvasion has already accomplished does not cause an evident clearance of PrP^Sc^ in the CNS, probably because they do not efficiently cross the blood-brain barrier or because their effective pharmacological concentrations *in vivo* need to be higher (Yun et al., 2007; Heiseke et al., 2010; Goold et al., 2015).

The molecular mechanisms that explain how autophagy protects against neurodegeneration have not yet been determined, although there are several hypotheses. In addition to reducing the basic toxic protein that causes the disease, autophagy has been proposed to eliminate damaged organelles, such as the mitochondria, and attenuate the apoptotic response to various forms of stress (Ravikumar et al., 2006; Zhu et al., 2007). In general, it is reasonable to assume that reducing PrP^Sc^ accumulation by stimulating autophagy could represent an effective therapeutic strategy for prion diseases in the near future, but additional and more complex studies are necessary.

## Conclusion

Although it is unknown whether the accumulation of autophagic vacuoles in the CNS of several neurodegenerative diseases reflects a proper response to deposition of misfolded proteins or an impaired autophagosome clearance, there is direct evidence indicating a decrease of autophagic activity or a deterioration of the lysosomal degradation process. Dysregulation of autophagy-related genes and proteins in various TSE models, along with the abnormal accumulation of autophagic vacuoles, fully supports the impairment of autophagy in prion diseases, which will probably impede the clearance of protein aggregates and damaged organelles from neurons and will contribute to prion replication, neurodegeneration, and the development of the disease. Altogether, research performed in this subject indicates that autophagy is involved in prion neuropathology *in vivo* and may be a potential target for therapeutic intervention. Unfortunately, the analysis of autophagy in TSE is currently incomplete. At the level of the whole organism, the therapeutic effectiveness of manipulating autophagy may depend on particular complex factors of the disease. More research involving appropriate experimental *in vivo* models, late preclinical animals, and different prion strains, will be necessary to earn more detailed information on the molecular mechanisms underlying autophagic impairment in prion diseases. Other challenges include clarifying whether dysregulated autophagy is a prerequisite or consequence of prion-induced toxicity, or whether increased autophagy may have deleterious effects. It is important to keep in mind that a certain level of autophagy may be a positive modifier of prion infection and susceptibility, so it cannot be ruled out that autophagy plays a dual role combining pro- and anti-prion effects. A better understanding of the role of autophagy in the specific conditions of prion infection and its consequences for neurodegeneration will benefit the development of innovative and effective therapeutic strategies based on the manipulation of this process.

## Author Contributions

ÓL-P collected the literature and drafted the original manuscript. RB, FL, and IM-B conceptualized, designed the study, and revised the content of the manuscript. JB and IF revised the manuscript critically for intellectual content. All authors read and approved the final version of the manuscript for publication.

## Conflict of Interest

The authors declare that the research was conducted in the absence of any commercial or financial relationships that could be construed as a potential conflict of interest.
